# Construction of a junction DNA nanostructure and modulation of the junction switching to quadruplexes

**DOI:** 10.1098/rsos.171337

**Published:** 2017-12-20

**Authors:** Yanwei Cao, Xiaoxuan Xiang, Renjun Pei, Yang Li, Yuting Yan, Xinhua Guo

**Affiliations:** 1 State Key Laboratory of Supramolecular Structure and Materials, College of Chemistry, Jilin University, Changchun 130012, People's Republic of China; 2 Key Laboratory for Molecular Enzymology and Engineering of the Ministry of Education, College of Life Science, Jilin University, Changchun 130012, People's Republic of China; 3 CAS Key Laboratory of Nano-Bio Interface, Division of Nanobiomedicine, Suzhou Institute of Nano-Tech and Nano-Bionics, Chinese Academy of Sciences, Suzhou 215123, People's Republic of China

**Keywords:** duplex junction, G-quadruplex, i-motif, DNA nanostructure, structural switch

## Abstract

A junction DNA nanostructure has been successfully built in lithium acetate buffer solution at a near-neutral pH value through the connection of two slipped junction structures that are formed by G-rich and C-rich strands. The GC-rich duplex junctions in the nanostructure can be switched to G-quadruplexes and i-motifs in weakly acidic potassium acetate solution, which leads to the assembly of DNA nanostructures composed of alternating quadruplex and duplex DNA structures. The transformation between different DNA nanoarchitectures may be applied to the operation of ‘DNA nanomachines’.

## 1. Introduction

DNA molecules are a promising material for the construction of nanoscale structures because of their low cost of synthesis, high specificity of assembly and conformational flexibility. In most cases, the DNA molecules are present as double helices linked by Watson–Crick hydrogen bonds within AT and GC base pairs. However, duplex DNA structures can be cleaved at certain sites by the invasion of homologous DNA strands, which leads to the formation of DNA helix junctions. Junction structures have been found to occur during the processes of gene conversion or genetic recombination [1,2]. They also serve as excellent templates for the construction of DNA supramolecules. For example, Seeman [3] established a stable four-stranded DNA junction, in which each strand contained a unique single-stranded sticky end for further linkage to various DNA junctions to form artificial two-dimensional lattices. Mao *et al.* [4] designed a parallelogram-shaped DNA junction and a triangular tile [5] consisting of four and three DNA junction units, respectively.

Furthermore, it is well known that a G-rich DNA strand containing tandem G-tracts can fold into a G-quadruplex, which is characterized by the stacking of G-quartets connected by cyclic Hoogsten hydrogen bonds, and specific cations (such as K^+^, Na^+^ and NH_4_^+^) can coordinate to the O_6_ atoms of the guanine residues to reduce the electrostatic repulsion and enhance the structural stability [6]. By contrast, a C-rich sequence containing several C-tracts may assemble into a four-stranded i-motif DNA scaffold under weakly acidic conditions; this structure is characterized by the intercalation of hemi-protonated cytosine–cytosine (C•CH^+^) base pairs [7]. Both G-quadruplexes and i-motifs are attracting increasing attention from researchers because the sequences that are able to form such structures can be found in many important regions of human chromosomes. The formation and dissociation of quadruplex DNA structures may play a significant role in physiological activities [8,9]. Interestingly, these structures are also widely used to build DNA nanostructures [10–12].

Our previous results demonstrated that the formation of quadruplex nanostructures can be manipulated by optimization of the DNA sequences and experimental conditions [13]. Meanwhile, it has been also reported that the structural switch between quadruplex and duplex DNA structures can be modulated by the solution pH value and cation species [14–16]. Notably, such transformations may be applied to the operation of ‘DNA machines’ [17,18] and ‘DNA logic gates’ [19,20].

**Figure 1.**
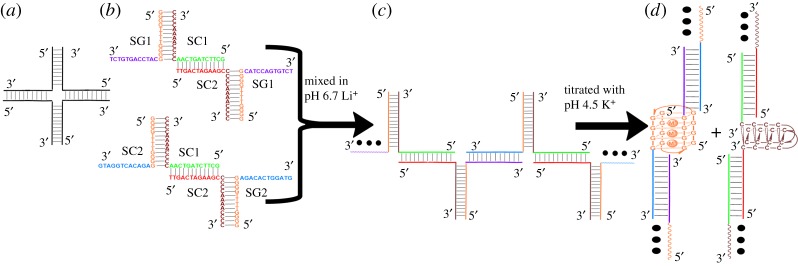
Schematic descriptions of (*a*) a four-way junction structure; (*b*) the designed slipped junction structures, DNA samples 1 and 2; (*c*) the DNA nanostructure assembled from two slipped junction structures in pH 6.7 lithium acetate solution, DNA sample 3; and (*d*) ‘duplex + quadruplex’-type DNA nanostructures generated from the structural switch of the junction DNA nanostructure in pH 4.5 potassium acetate solution, DNA sample 4 that contains two kinds of newly generated nanostructures: ‘duplex + i-motif’-type (DNA sample 5) and ‘duplex + G-quadruplex’-type DNA nanostructures (DNA sample 6).

In this work, based on the four-way junction structure illustrated in figure 1*a*, we have built a slipped four-way junction structure by mixing two C-rich DNA sequences, 5′-GCTTCTAGTCAA**C_4_A_4_C_4_**-3′ (SC1) and 5′-TTGACTAGAAGC**C_4_A_4_C_4_**-3′ (SC2), with a G-rich sequence, 5′-**G_4_T_4_G_4_**CATCCAGTGTCT-3′ (SG1), in a 1 : 1 : 2 ratio in lithium acetate solution at pH 6.7, which generated two single-stranded flexible branches from SG1 (figure 1*b*, top). Similarly, the two C-rich sequences SC1 and SC2 were mixed with another G-rich sequence 5′-**G_4_T_4_G_4_**AGACACTGGATG-3′ (SG2) in the same way to build a slipped four-way junction structure with two single-stranded arms from SG2 (figure 1*b*, bottom). The G-/C-rich linker regions of the selected DNA strands are shown in bold font. Finally, a mixture of the two slipped junction structures could assemble to form a junction nanostructure through interaction of the unpaired arms of SG1 and SG2 (figure 1*c*). Note that the GC-rich duplex parts of the assembled nanostructure could be dissociated under specific solution conditions, in which case the G-rich parts folded into bimolecular G-quadruplexes and the C-rich parts assembled into bimolecular i-motifs. It has been reported that the tetraplex structure of d(G_4_T_4_G_4_) that is formed in either K^+^ or Na^+^ solution shows the same folding topology with two diagonal T_4_ loops [21]. K^+^ cations were used here because UV melting experiments demonstrated that the antiparallel G-quadruplex structures formed by d(G_4_T_4_G_4_) are more stable in K^+^ solution than in Na^+^ solution (75°C versus 52°C), as shown in the electronic supplementary material, figure S1. According to the previous results, the assembly of bimolecular quadruplexes with duplex linkage units could form one-dimensional DNA nanostructures composed of alternating quadruplex and duplex DNA structures (figure 1*d*) [22,23]. However, different lengths of T-spacers between the double-stranded DNA and i-motif structures may cause the formation of different ‘closed’ structures [23]. To simplify the analysis, the T-spacers were removed from our designed sequences.

## 2. Material and methods

### 2.1. Material and sample preparation

The oligonucleotides (OPC grade) were synthesized by TaKaRa Biotechnology Co., Ltd. (Dalian, China) and used as received. The DNA fragments studied here are listed below.

5′-GCTTCTAGTCAAC_4_A_4_C_4_-3′ (SC1) Mr = 7183.25;
5′-TTGACTAGAAGCC_4_A_4_C_4_-3′ (SC2) Mr = 7232.26;
5′-G_4_T_4_G_4_CATCCAGTGTCT-3′ (SG1) Mr = 7443.24;
5′-G_4_T_4_G_4_AGACACTGGATG-3′ (SG2) Mr = 7541.27.

Stock solutions of the oligonucleotides were prepared by directly dissolving the lyophilized powder in Milli-Q water (MA, USA) to approximately 2 mM and were stored at −18°C. Accurate concentrations of the oligonucleotides were obtained from their UV absorbances at 260 nm at 90°C using molar absorptivity tabulated at the following website: http://www.idtdna.com/calc/analyzer. Lithium acetate and potassium acetate buffer solutions (pH 6.7) were prepared by directly dissolving the lithium acetate solid and potassium acetate solid in the Milli-Q water; the potassium acetate buffer solution with pH value of 4.5 was obtained by adding acetic acid.

For the DNA assembly, different oligomers (50 µM of each oligomer) were mixed in pH 6.7 (50 mM Li^+^) or pH 4.5 (100 mM K^+^) buffer solution in different ratios. DNA samples were heated in a 90°C water bath for 10 min and slowly cooled to room temperature (about 10 h), followed by equilibration at 4°C for more than four days. For the DNA samples that needed further reforming: after the mixing or titration process, these DNA samples were heated at 30°C for 4 h and slowly cooled to room temperature, followed by equilibration at 4°C for more than 4 days.

### 2.2. UV absorption spectrophotometry

UV melting curves were recorded on a UV-2550 spectrophotometer (SHIMADZU, Kyoto, Japan) fitted with an S-1700 temperature controller measured at 295 nm as in previous reports [24,25]. The temperature was increased at a heating rate of 0.5°C min with DNA samples being left to equilibrate for 0.5 min at each temperature. The absorption cell was sealed to prevent solvent evaporation, and the bubble was removed by stirring during the heating process.

### 2.3. Circular dichroism spectroscopy

Circular dichroism (CD) spectra were measured on a J-810 CD spectrometer (JASCO, Tokyo, Japan) using a 0.02 cm path length Hellma cell at 20°C. Each DNA spectrum corresponded to the average of three scans with a scan range from 200 to 320 nm. Each trace was obtained at 100 nm min^−1^ of scanning speed, with response time of 2 s, data pitch of 1 nm and bandwidth of 1 nm. The background spectra containing the buffer alone were deducted from all DNA spectra.

### 2.4. Native gel electrophoresis

The 8% poly/acrylamide gel was used for the native electrophoresis analysis and run at 4°C. 1× TBE and 2-(*N*-morpholino) ethanesulfonic acid monohydrate (MES, pH 4.5, 50 mM) buffer solutions were used to prepare the neutral and acid gels, respectively. The running buffers were further supplemented with 50 mM LiOAc for the neutral gel and 100 mM KOAc for the acid gel. The gel electrophoresis was performed for 3.5 h at 100 V. Results were observed using an Ultrapower™ visible light transilluminator (Bioteke, Beijing, China) after using GelGreen dye (Biotium) in 100 mM NaCl. The gel image was processed with Adobe Photoshop CS6 software to reduce background interference [13].

### 2.5. Atomic force microscopy

Atomic force microscopy (AFM) experiments were performed on mica surfaces that can bind nucleic acids due to the adsorption of magnesium ions [26]. Here, the DNA samples (50 µM of each DNA) were annealed in different buffers containing 50 mM LiOAc or 100 mM KOAc at 4°C. Then, aliquots were diluted with 4 mM MgCl_2_ aqueous solution to give a 20 µl analyte containing 5 µM DNA strands. The analytes were equilibrated on freshly cleaved mica plates for 5–8 min. After that, excess fluid was removed. Then, the mica plates were swilled with Milli-Q water carefully and repeatedly, and left to air-dry, in a very clean container to avoid dust settling. AFM analysis was in tapping mode with a Nanoscope IIIa scanning probe microscope (Bruker) from Digital Instruments by utilizing NANOSENSORS™ PPP-NCHR AFM probes. The AFM images were enhanced by flattening to remove the background slope and were modulated for contrast and brightness.

## 3. Results and discussion

### 3.1. The structural characteristics of various DNA conformations studied with circular dichroism experiments

**Figure 2.**
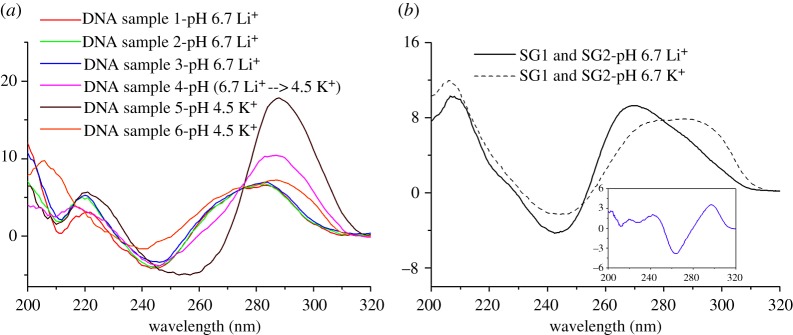
CD spectra of different oligonucleotides (50 µM of each oligomer) annealed under different conditions. (*a*) DNA samples 1 and 2: the mixtures of SC1, SC2 and SG1 and of SC1, SC2 and SG2 in ratios of 1 : 1 : 2 in 50 mM pH 6.7 LiOAc buffer solutions indicated by red and green solid lines, respectively; DNA sample 3: the mixture of DNA samples 1 and 2 in a 1 : 1 ratio (blue solid line); DNA sample 4: DNA sample 3 is titrated with pH 4.5 KOAc buffer solution and the final concentration of K^+^ is 100 mM (magenta solid line); DNA samples 5 and 6: the mixtures of SC1 and SC2 and of SG1 and SG2 in a 1 : 1 ratio in 100 mM pH 4.5 KOAc buffer solutions indicated by wine and orange solid lines, respectively; (*b*) the mixtures of SG1 and SG2 in a 1 : 1 ratio annealed in 100 mM pH 6.7 LiOAc and pH 6.7 KOAc buffer solutions indicated by black solid and dashed lines, respectively; inset: differential CD spectrum acquired by deducting the spectrum obtained in Li^+^ from the one obtained in K^+^ solution.

The CD experiments were performed first to reveal the structural features of the designed junction conformations and the structural transitions. Figure 2*a* shows the CD spectra of various DNA strands mixed under different solution conditions. DNA samples 1 and 2 were obtained by mixing strands SC1, SC2 and SG1 and strands SC1, SC2 and SG2, respectively, in ratios of 1 : 1 : 2 in 50 mM LiOAc buffer (pH 6.7); DNA sample 3 was obtained by mixing samples 1 and 2. All three junction DNA structures (samples 1, 2 and 3) give a positive band at around 280 nm and a negative band at around 240 nm in their CD spectra, which indicates that the majority of DNA structures in the three samples are duplexes [27]. However, the CD signal exhibits an obvious enhancement at around 290 nm when DNA sample 3 is titrated with pH 4.5 KOAc buffer; this result is suggestive of a structural shift from the duplex junction to the four-stranded DNA conformation containing bimolecular i-motifs and antiparallel G-quadruplexes (figure 1*d*) [27]. This is consistent with the previous report that quadruplexes can be generated from GC duplexes [16].

Additionally, compared with the aforementioned spectra, the CD spectrum of DNA sample 5 (prepared by mixing SC1 and SC2 in a 1 : 1 ratio in 100 mM pH 4.5 KOAc buffer solution) displays positive bands at around 220 nm and 290 nm and a negative band at around 265 nm, which confirms that an i-motif structure is formed under weakly acidic conditions. By contrast, the CD spectrum of DNA sample 6 (obtained by mixing SG1 and SG2 in a 1 : 1 ratio in 100 mM pH 4.5 KOAc buffer solution) exhibits positive bands at around 220 nm and 290 nm and a negative band at around 240 nm. Although this spectrum shows some differences from the characteristic peaks of antiparallel G-quadruplexes [27], the differential CD spectrum acquired by the deduction of the CD spectrum obtained in Li^+^ solution from the one obtained in K^+^ solution (figure 2*b*, inset) displays positive bands at around 290 nm and 240 nm and a negative band at around 260 nm, which confirms the formation of antiparallel G-quadruplexes upon mixing of the two G-rich strands [22]. The bimolecular quadruplexes associated with two C-strands and two G-strands are linked by double-helix DNA structures, so a new type of DNA nanostructure containing alternating duplex and quadruplex structures can be formed.

### 3.2. The formation of DNA nanostructures confirmed by native-polyacrylamide gel electrophoresis experiments

**Figure 3.**
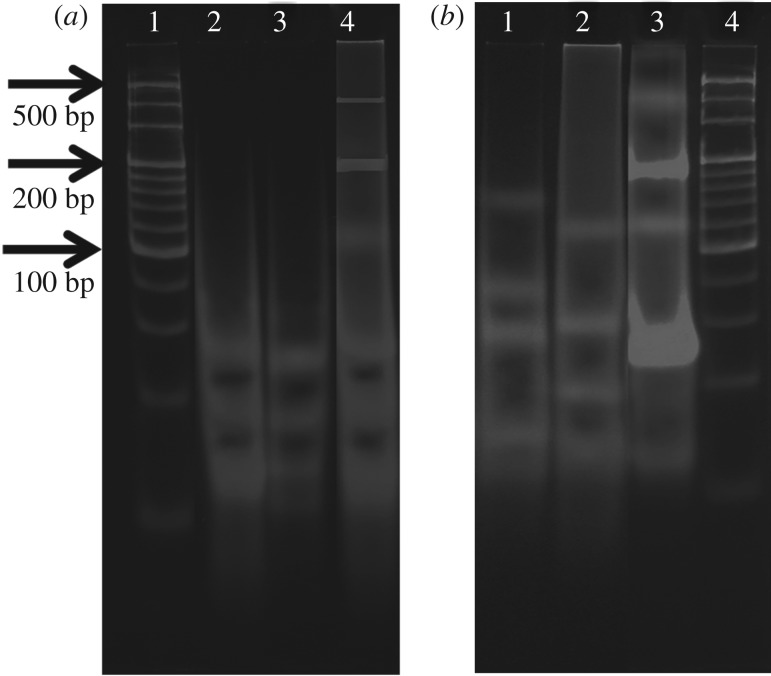
8% Native-PAGE images of the aforementioned DNA samples. (*a*) Lane 1: the molecular mass ladder with 20 bp step ladder from 20 bp to 200 bp and 100 bp step ladder from 200 bp to 500 bp; lanes 2, 3 and 4 correspond to DNA samples 1, 2 and 3, respectively; (*b*) lane 1: DNA sample 5; lane 2: DNA sample 6; lane 3: DNA sample 4; lane 4: the molecular mass ladder.

Native-polyacrylamide gel electrophoresis (PAGE) experiments were performed in 50 mM pH 6.7 LiOAc and 100 mM pH 4.5 KOAc buffer solutions for the above-mentioned DNA samples to confirm the formation of the DNA nanostructures. As shown in figure 3*a*, DNA samples 1 and 2 both exhibit three obvious bands in lanes 2 and 3. According to the electrophoretic mobility of the molecular mass ladder in lane 1, the three bands correspond to single-stranded, double-helix and slipped junction DNA structures formed in mixtures of SC1, SC2 and SG1 and of SC1, SC2 and SG2. By comparison, DNA sample 3 in lane 4 mainly exhibits slower migrating bands, including an obvious band at around 200 bp and other smear bands; this indicates the formation of DNA nanostructures assembled from the two slipped junction structures. Figure 3*b* shows that DNA samples 5 and 6, in lanes 1 and 2, not only exhibit bands at around 40 bp and 60 bp but also exhibit some slower migrating bands. These results manifest the formation of ‘duplex + quadruplex’-type DNA nanostructures in the 1 : 1 mixtures of two C-rich strands and two G-rich strands, which is in accordance with the reports in the literature [22,23]. However, in comparison with lane 4 in figure 3*a*, lane 3 in figure 3*b* exhibits more varied components, including bands at around 60 bp, 100 bp, 200 bp and several smear bands at the top of the gel, which indicates the existence of more types of DNA nanostructure after the addition of H^+^ and K^+^ to DNA sample 3. We propose that these components include the junction DNA nanostructures and ‘duplex + quadruplex’-type DNA nanostructures generated by the conversion from GC-rich duplexes to quadruplex structures.

### 3.3. Assembly of various types of DNA nanostructures characterized by atomic force microscopy experiments

**Figure 4.**
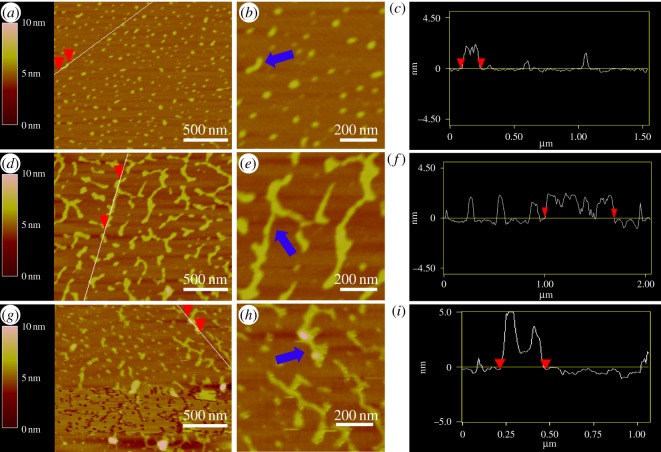
(*a*), (*d*) and (*g*) AFM images with the scale bar of 500 nm corresponding to DNA samples 1, 3 and 4, respectively; the labelled DNA structures in (*a*), (*d*) and (*g*) were enlarged and indicated with the blue arrow in (*b*), (*e*) and (*h*), respectively; (*c*), (*f*) and (*i*) the height and length profiles of the DNA samples 1, 3 and 4, respectively.

Furthermore, AFM experiments were carried out to provide additional evidence for the assembly of the designed structures. As shown in figure 4*a,b*, several small ellipsoids and short bar-shaped aggregates are observed for DNA sample 1. The height of the aggregates is 1.5 nm on average (figure 4*c*), which indicates that these structures are composed of double helixes [28,29]. Because the designed junction structure contains two single-stranded branches and the junction sites are free to rotate, these bar-shaped aggregates with an average length of 150 nm are consistent with our designed slipped junction structures. As expected, many larger-sized aggregates are observed for DNA sample 3. These aggregates with several short branches, as shown in figure 4*d,e*, are in accordance with DNA nanostructures assembled from two slipped junction structures. Figure 4*f* shows that the lengths of these nanostructures are capable of reaching 700 nm in one direction. After DNA sample 3 was titrated with pH 4.5 KOAc buffer, some rod-shaped aggregates occurred, as shown in figure 4*g,h*. After the analysis of these new structures, we found that the measured heights of these nanoparticles are around 4.0 nm (figure 4*i*). In accordance with previous reports [13,22,23], these particles are DNA nanostructures containing quadruplex DNA structures and the aggregates with higher diameters may be due to typical lamellar stacking of different DNA structures.

**Figure 5.**
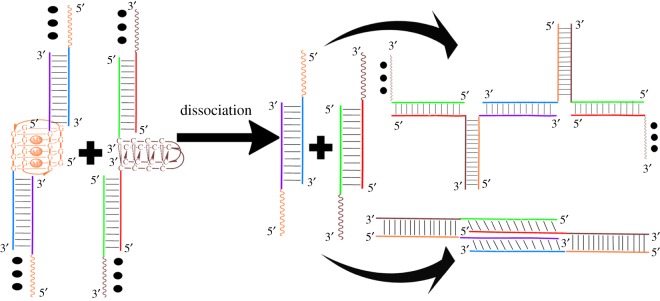
The possibility of structural switch from ‘duplex + quadruplex’-type DNA nanostructures to junction DNA nanostructures; two types of structures may be formed: the junction DNA nanostructure (right top) and ‘closed’ duplex DNA structure (right bottom).

We also studied the possibility of structural switch from the ‘duplex + quadruplex’-type DNA nanostructures to junction DNA nanostructures, as shown in figure 5. The i-motif and G-quadruplex structures stabilized by K^+^ ions can be dissociated in an alkaline environment [23] and with the addition of 18-crown-6-ether [30], respectively. In this study, to simplify the experiment, we directly mixed the two C-rich and two G-rich strands, respectively, in a ratio of 1 : 1 in 50 mM LiOAc buffer (pH 6.7). The mixtures were annealed for four days and then further mixed, as DNA samples 1 and 2 were. Based on the above method, native-PAGE and AFM experimental results indicated that more species of around 60 bp are formed and the yield of mismatched DNA nanostructures is less than that in DNA sample 3 (electronic supplementary material, figure S2). This may be due to the formation of interlocked duplexes or other complex DNA structures (figure 5, right bottom).

## 4. Conclusion

To summarize, we have proposed a new type of DNA monomer structure based on four-way junction structures. Our results demonstrate that these monomers can assemble into stable junction DNA nanostructures, as confirmed by CD spectroscopy, native-PAGE and AFM experiments. Importantly, the junction nanostructures have the ability to transform into another type of DNA nanostructure consisting of alternating duplex and quadruplex DNA scaffolds; this transformation can be modulated by the solution pH value and the nature of the monovalent cations that are present. Such transformations show great potential for the operation of ‘DNA nanomachines’. Notably, the slipped junction structures could be formed with only three DNA strands and the construction of the junction DNA nanostructure consumes four DNA strands in total if the GC-rich duplex arms in the nanostructures are kept the same. However, this may also induce the formation of interlocked duplex DNA structures, which results in low structural conversion efficiency from the ‘duplex + quadruplex’-type DNA nanostructures to the junction DNA nanostructures. Our previous results have revealed that G- or C-rich strands can undergo ‘self-assembly’ in the formation of quadruplex structures by altering the length of the G- or C-repeats [31]. Following on from this work, we will optimize the nature of the G- and C-rich strands to improve the operation mechanism of the potential ‘DNA nanomachine’. Furthermore, the formation and dissociation of the quadruplex DNA structures would be more easily controlled by regulating the solution pH value and by the addition and removal of divalent cations, in accordance with previous methods [17,32]; this would allow the operation of the ‘DNA nanomachine’ in a circular pattern.

## Supplementary Material

Figure S1;Figure S2

## Data accessibility

The datasets supporting this article have been uploaded as part of the electronic supplementary material.

## References

[1] Holliday R 1964 A mechanism for gene conversion in fungi. Gen. Res. 5, 282–304. (doi:10.1017/S0016672300001233) 10.1017/S0016672308009476 18976517

[2] Broker TR, Lehman IR 1971 Branched DNA molecules: intermediates in T4 recombination. J. Mol. Biol. 60, 131–149. (doi:10.1016/0022-2836(71)90453-0) 4937189 10.1016/0022-2836(71)90453-0

[3] Seeman NC 2003 DNA in a material world. Nature 421, 427–431. (doi:10.1038/nature01406) 12540916 10.1038/nature01406

[4] Mao C, Weiqiong Sun A, Seeman NC 1999 Designed two-dimensional DNA holliday junction arrays visualized by atomic force microscopy. J. Am. Chem. Soc. 121, 5437–5443. (doi:10.1021/ja9900398)

[5] Liu D, Wang M, Deng Z, Walulu R, Mao C 2004 Tensegrity: construction of rigid DNA triangles with flexible four-arm DNA junctions. J. Am. Chem. Soc. 126, 2324–2325. (doi:10.1021/ja031754r) 14982434 10.1021/ja031754r

[6] Huppert JL 2008 Four-stranded nucleic acids: structure, function and targeting of G-quadruplexes. Chem. Soc. Rev. 37, 1375–1384. (doi:10.1039/b702491f) 18568163 10.1039/b702491f

[7] Guéron M, Leroy J-L 2000 The i-motif in nucleic acids. Curr. Opin. Struct. Biol. 10, 326–331. (doi:10.1016/S0959-440X(00)00091-9) 10851195 10.1016/s0959-440x(00)00091-9

[8] Lipps HJ, Rhodes D 2009 G-quadruplex structures: *in vivo* evidence and function. Trends Cell Biol. 19, 414–422. (doi:10.1016/j.tcb.2009.05.002) 19589679 10.1016/j.tcb.2009.05.002

[9] Dembska A 2016 The analytical and biomedical potential of cytosine-rich oligonucleotides: a review. Anal. Chim. Acta 930, 1–12. (doi:10.1016/j.aca.2016.05.007) 27265899 10.1016/j.aca.2016.05.007

[10] Davis JT 2004 G-quartets 40 years later: from 5′-GMP to molecular biology and supramolecular chemistry. Angew. Chem. Int. Ed. 43, 668–698. (doi:10.1002/anie.200300589) 10.1002/anie.200300589 14755695

[11] Alberti P, Bourdoncle A, Sacca B, Lacroix L, Mergny JL 2006 DNA nanomachines and nanostructures involving quadruplexes. Org. Biomol. Chem. 4, 3383–3391. (doi:10.1039/b605739j) 17036128 10.1039/b605739j

[12] Dong Y, Yang Z, Liu D 2014 DNA nanotechnology based on i-motif structures. Acc. Chem. Res. 47, 1853–1860. (doi:10.1021/ar500073a) 24845472 10.1021/ar500073a

[13] Cao Y, Gao S, Yan Y, Bruist MF, Wang B, Guo X 2017 Assembly of supramolecular DNA complexes containing both G-quadruplexes and i-motifs by enhancing the G-repeat-bearing capacity of i-motifs. Nucleic Acids Res. 45, 26–38. (doi:10.1093/nar/gkw1049) 27899568 10.1093/nar/gkw1049 PMC5224476

[14] Li W, Wu P, Ohmichi T, Sugimoto N 2002 Characterization and thermodynamic properties of quadruplex/duplex competition. FEBS Lett. 526, 77–81. (doi:10.1016/S0014-5793(02)03118-6) 12208508 10.1016/s0014-5793(02)03118-6

[15] Phan AT, Mergny JL 2002 Human telomeric DNA: G-quadruplex, i-motif and Watson-Crick double helix. Nucleic Acids Res. 30, 4618–4625. (doi:10.1093/nar/gkf597) 12409451 10.1093/nar/gkf597 PMC135813

[16] Miyoshi D, Matsumura S, Li W, Sugimoto N 2003 Structural polymorphism of telomeric DNA regulated by pH and divalent cation. Nucleic Acids Res. 22, 203–221. (doi:10.1081/NCN-120019528) 10.1081/NCN-120019528 12744606

[17] Liu D, Balasubramanian S 2003 A proton-fuelled DNA nanomachine. Angew. Chem. Int. Ed. 42, 5734–5736. (doi:10.1002/anie.200352402) 10.1002/anie.200352402 14661209

[18] Alberti P, Mergny JL 2003 DNA duplex-quadruplex exchange as the basis for a nanomolecular machine. Proc. Natl Acad. Sci. USA 100, 1569–1573. (doi:10.1073/pnas.0335459100) 12574521 10.1073/pnas.0335459100 PMC149873

[19] Miyoshi D, Inoue M, Sugimoto N 2006 DNA logic gates based on structural polymorphism of telomere DNA molecules responding to chemical input signals. Angew. Chem. Int. Ed. 45, 7716–7719. (doi:10.1002/anie.200602404) 10.1002/anie.200602404 17031891

[20] Li T, Zhang L, Ai J, Dong S, Wang E 2011 Ion-tuned DNA/Ag fluorescent nanoclusters as versatile logic device. ACS Nano 5, 6334–6338. (doi:10.1021/nn201407h) 21732637 10.1021/nn201407h

[21] Schultze P, Hud NV, Smith FW, Feigon J 1999 The effect of sodium, potassium and ammonium ions on the conformation of the dimeric quadruplex formed by the *Oxytricha nova* telomere repeat oligonucleotide d(G(4)T(4)G(4)). Nucleic Acids Res. 27, 3018–3028. (doi:10.1093/nar/27.15.3018) 10454595 10.1093/nar/27.15.3018 PMC148525

[22] Dutta K, Fujimoto T, Inoue M, Miyoshi D, Sugimoto N 2010 Development of new functional nanostructures consisting of both DNA duplex and quadruplex. Chem. Commun. 46, 7772–7774. (doi:10.1039/c0cc00710b) 10.1039/c0cc00710b 20820501

[23] Ren J, Wang T, Wang E, Wang J 2016 I-motif-stapled and spacer-dependent multiple DNA nanostructures. RSC Adv. 6, 87 021–87 025. (doi:10.1039/C6RA15201E)

[24] Mergny J-L, Phan A-T, Lacroix L 1998 Following G-quartet formation by UV-spectroscopy. FEBS Lett. 435, 74–78. (doi:10.1016/S0014-5793(98)01043-6) 9755862 10.1016/s0014-5793(98)01043-6

[25] Mergny JL, Lacroix L 2009 UV melting of G-quadruplexes. Curr. Protoc. Nucleic Acid Chem. 20, 17.1. 1–17.1. 15. (doi:10.1002/0471142700.nc1701s37) 10.1002/0471142700.nc1701s37 19488970

[26] Thomson NH 2007 Atomic force microscopy of DNA structure and interactions. Berlin, Germany: Springer.

[27] Kypr J, Kejnovská I, Renčiuk D, Vorlíčková M 2009 Circular dichroism and conformational polymorphism of DNA. Nucleic Acids Res. 37, 1713–1725. (doi:10.1093/nar/gkp026) 19190094 10.1093/nar/gkp026 PMC2665218

[28] Morenoherrero F, Colchero J, Baró AM 2003 DNA height in scanning force microscopy. Ultramicroscopy 96, 167–174. (doi:10.1016/S0304-3991(03)00004-4) 12672567 10.1016/S0304-3991(03)00004-4

[29] Adamcik J, Klinov DV, Witz G, Sekatskii SK, Dietler G 2006 Observation of single-stranded DNA on mica and highly oriented pyrolytic graphite by atomic force microscopy. FEBS Lett. 580, 5671–5675. (doi:10.1016/j.febslet.2006.09.017) 17007844 10.1016/j.febslet.2006.09.017

[30] Lu CH, Cecconello A, Willner I 2016 Recent advances in the synthesis and functions of reconfigurable interlocked DNA nanostructures. J. Am. Chem. Soc. 138, 5172–5185. (doi:10.1021/jacs.6b00694) 27019201 10.1021/jacs.6b00694

[31] Cao Y, Gao S, Li C, Yan Y, Wang B, Guo X 2016 Structural varieties of selectively mixed G- and C-rich short DNA sequences studied with electrospray ionization mass spectrometry. J. Mass Spect. 51, 931–937. (doi:10.1002/jms.3804) 10.1002/jms.3804 27378414

[32] Fahlman RP, Hsing M, Sporer-Tuhten CS, Sen D 2003 Duplex pinching: a structural switch suitable for contractile DNA nanoconstructions. Nano Lett. 3, 1073–1078. (doi:10.1021/nl034267i)

