# Supplementary material for: Construction of a junction DNA nanostructure and modulation of the junction switching to quadruplexes
Source: R Soc Open Sci. 2017 Dec 20;4(12):171337. doi: 10.1098/rsos.171337 (PMC5750025; doi:10.1098/rsos.171337)
Supplement: Figure S1;Figure S2 [file rsos171337supp1.doc]

**Supporting information**

**Construction of a junction DNA nanostructure and modulation of the junction switching to quadruplexes**

Yanwei Cao^1^, Xiaoxuan Xiang^1^, Renjun Pei^3^, Yang Li^1^, Yuting Yan^1^ and Xinhua Guo^*1, 2^

^1^State Key Laboratory of Supramolecular Structure and Materials, College of Chemistry, Jilin University, Changchun 130012, P.R. China

^2^Key Laboratory for Molecular Enzymology and Engineering of the Ministry of Education, College of Life Science, Jilin University, Changchun130012, P.R. China

^3^CAS Key Laboratory of Nano-Bio Interface, Division of Nanobiomedicine, Suzhou Institute of Nano-Tech and Nano-Bionics, Chinese Academy of Sciences, Suzhou 215123, China

Address reprint requests to:

Prof. Xinhua Guo

Address: 2699 Qianjin Street, Changchun China, 130012

Phone number: 86-431-89228949

Fax number: 86-431-89228949

Email: [guoxh@jlu.edu.cn](mailto:guoxh@jlu.edu.cn)

**
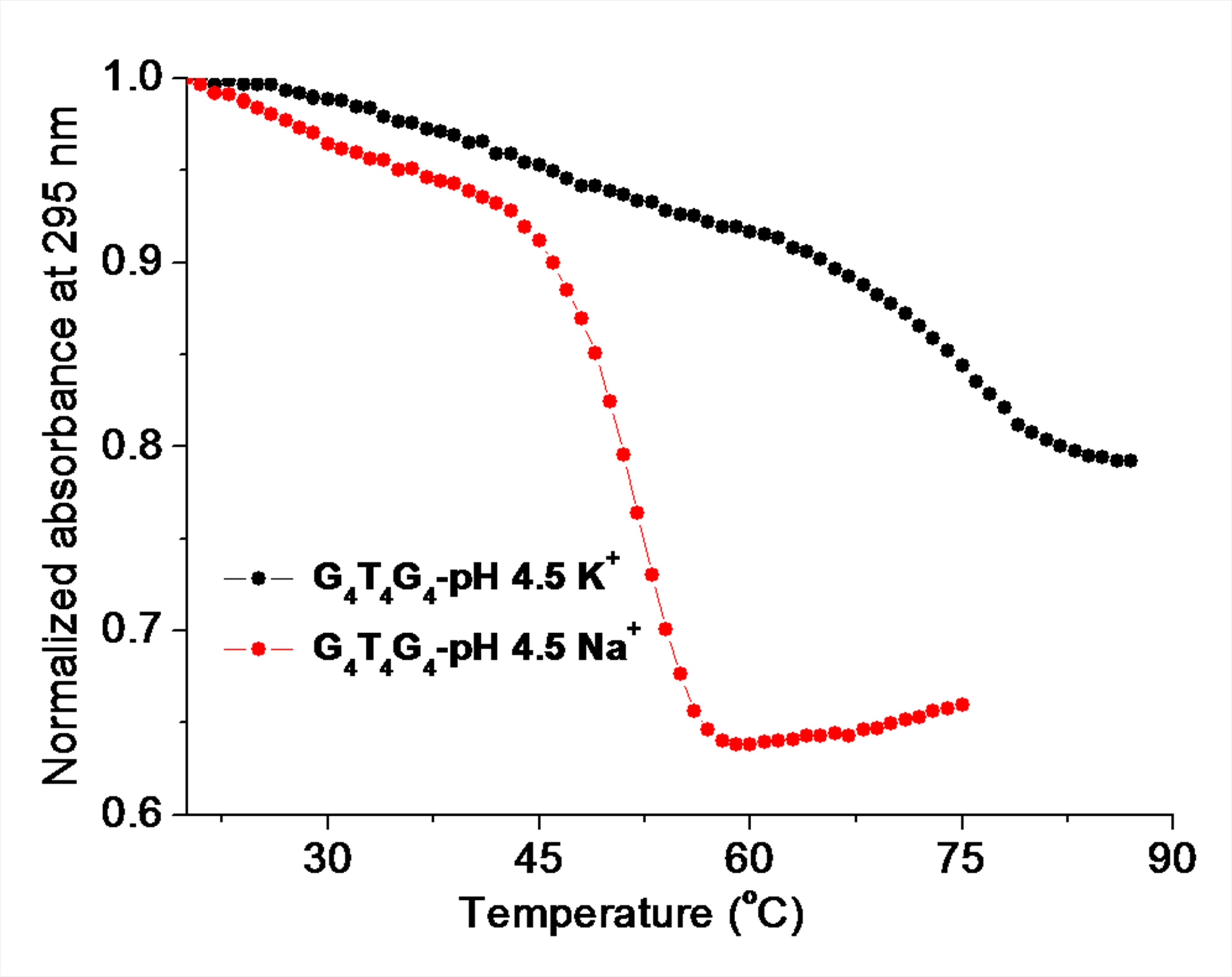
**

**Figure S1.** Normalized UV melting curves of d(G_4_T_4_G_4_) at 295 nm annealed in pH 4.5 buffer solutions with 100 mM K^+^ or 100 mM Na^+^ indicated by black and red lines, respectively; the final DNA concentration is 20 μM for UV melting experiments.


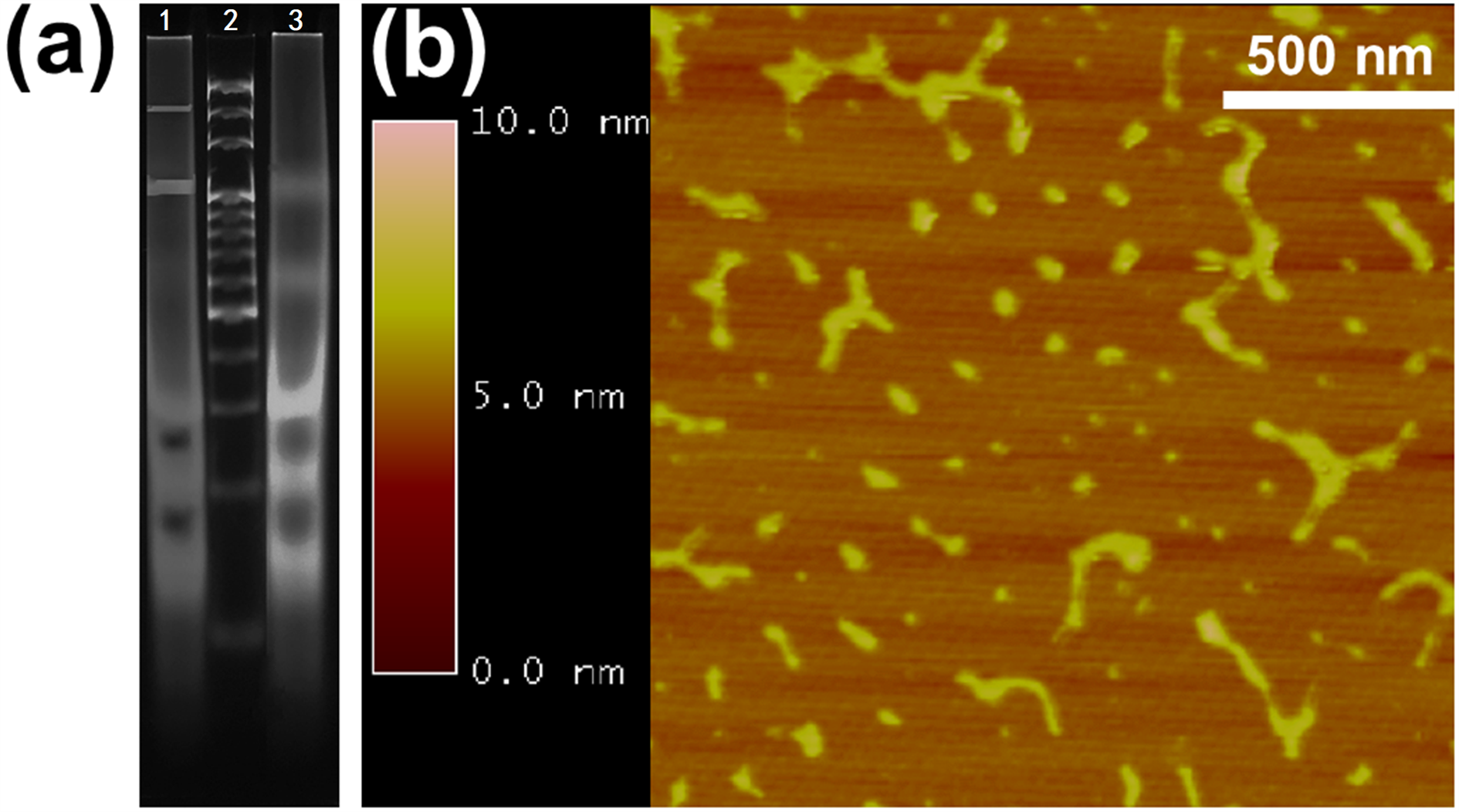


**Figure S2.** (a) 8% native PAGE image of junction DNA nanostructures, lane 1: DNA sample 3, lane 2: the molecular mass ladder, lane 3: DNA sample 7 (the 1: 1 mixtures of two C-rich and two G-rich DNA strands were annealed separately in 50 mM LiOAc buffer at pH 6.7 for four days, and then the two DNA samples were further mixed and equilibrated using the method for preparing DNA sample 3); (b) the AFM image with the scale bar of 500 nm corresponding to DNA sample 7
